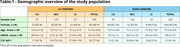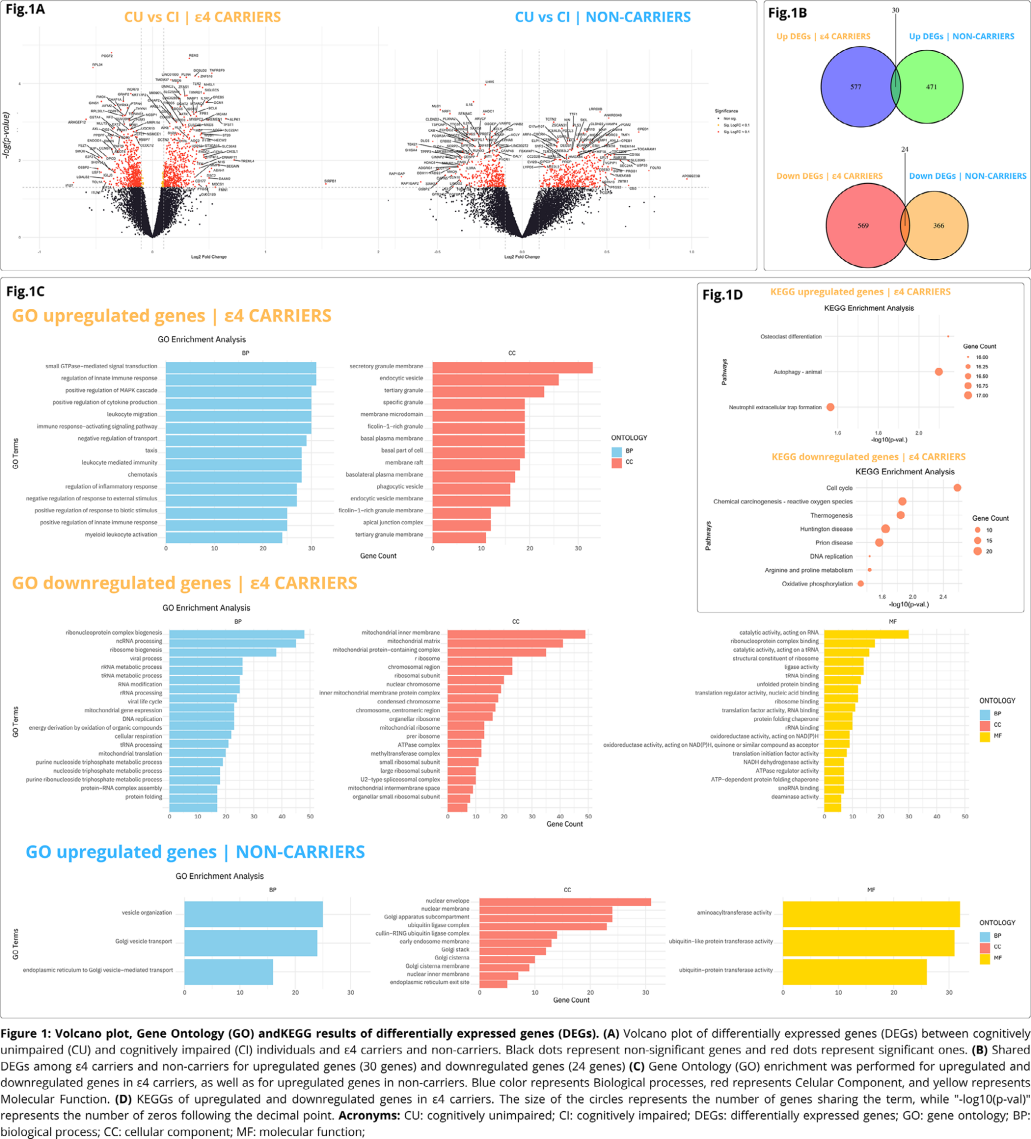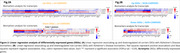# Influence of APOEε4 on Blood Transcriptomics in Cognitively Impaired Individuals

**DOI:** 10.1002/alz70862_110084

**Published:** 2025-12-23

**Authors:** Marcelo Madrid de Bittencourt, Gabriela Mantovani Baldasso, Marco Antônio De Bastiani, Christian Limberger, João Batista Teixeira da Rocha, Diogo O. Souza, Eduardo R. Zimmer

**Affiliations:** ^1^ Universidade Federal do Rio Grande do Sul, Porto Alegre, Rio Grande do Sul Brazil; ^2^ Brain Institute of Rio Grande do Sul, Porto Alegre Brazil; ^3^ McGill Centre for Studies in Aging, Montreal, QC Canada

## Abstract

**Background:**

The APOEε4 allele is a well‐known genetic risk factor for sporadic Alzheimer's disease (AD). However, its influence on gene expression in peripheral blood remains underexplored. This study aimed to investigate the influence of the ε4 allele on blood gene expression in cognitively unimpaired (CU) and impaired (CI) individuals.

**Method:**

We selected 423 individuals from the ADNI database with available APOE status, clinical diagnosis, and blood microarray data. CU and CI individuals were divided into ε4 carriers (*n* = 184) and non‐carriers (*n* = 239) (Table.1). Differentially expressed genes (DEGs) between CU and CI individuals stratified by APOEε4 carriership were analyzed in R using the Limma package. Linear models were performed to associate DEGs with CSF Aβ42, pTau181, total‐Tau, and FDG‐PET (*p* <0.05). In addition, pathway enrichment analysis using Gene Ontology (GO) and KEGG was performed for up and downregulated genes across groups.

**Results:**

The CU vs. CI comparison identified 607 upregulated and 593 downregulated genes in carriers, while non‐carriers showed 501 upregulated and 390 downregulated genes (Figure 1A‐B). In the enrichment analyses, carriers revealed upregulated pathways related to innate immunity, neutrophil degranulation, MAPK cascade, and GTPase signaling, while downregulated pathways were associated with ribosome biogenesis, mitochondrial function, cell cycle regulation, and RNA metabolism. For non‐carriers, only upregulated pathways showed significant enrichment, primarily in ubiquitin‐related processes and Golgi vesicle transport and organization (Figure 1B‐C). Linear regressions identified 106 upregulated genes (70 carriers, 36 non‐carriers) and 79 downregulated genes (55 carriers, 24 non‐carriers) influencing at least one biomarker (CSF Aβ42, pTau181, total‐Tau, or FDG‐PET). Among carriers, FDG‐PET emerged as a notable biomarker, showing a negative association with most upregulated genes (60) and a positive association with most downregulated genes (39). Conversely, Aβ42 was the most altered biomarker in non‐carriers, following a similar pattern to FDG‐PET in carriers, being negatively associated with 31 upregulated genes and positively associated with 15 downregulated genes (Figure 2A‐B).

**Conclusion:**

Our findings suggest the APOEε4 allele influences blood gene expression in CI individuals, leading to distinct transcriptomic signatures and pathophysiological pathways. Additionally, distinct gene expression patterns in carriers and non‐carriers impact biomarker variability, notably FDG‐PET and Aβ42.